# Roles of retinoic acid-inducible gene-I-like receptors (RLRs), Toll-like receptor (TLR) 3 and 2′-5′ oligoadenylate synthetase as viral recognition receptors on human mast cells in response to viral infection

**DOI:** 10.1007/s12026-014-8617-x

**Published:** 2014-12-31

**Authors:** Mizuho Tsutsui-Takeuchi, Hiroko Ushio, Minoru Fukuda, Takahiko Yamada, François Niyonsaba, Ko Okumura, Hideoki Ogawa, Shigaku Ikeda

**Affiliations:** 1 Department of Dermatology, Juntendo University School of Medicine, Bunkyo-ku, Tokyo 113-8421 Japan; 2 Atopy (Allergy) Research Center, Juntendo University School of Medicine, 2-1-1 Hongo, Bunkyo-ku, Tokyo 113-8421 Japan; 3 Department of Infection Control Science, Juntendo University School of Medicine, Bunkyo-ku, Tokyo 113-8421 Japan

**Keywords:** Mast cells, Virus, Retinoic acid-inducible gene-I-like receptors, Toll-like receptors, 2′-5′ Oligoadenylate synthetase

## Abstract

To investigate the anti-viral responses of human mast cells, we performed PCR array analysis of these cells after infection with vesicular stomatitis virus (VSV). PCR array analysis revealed that human mast cells up-regulated several anti-viral genes, including melanoma differentiation-associated gene 5, retinoic acid-inducible gene-I, and Toll-like receptor 3, together with type I interferons and chemokines, upon VSV infection. Additionally, we found that 2′-5′ oligoadenylate synthetase, which also works as a virus recognition receptor by activating the latent form of RNase L, leading to viral RNA degradation, was up-regulated in human mast cells upon VSV infection. Moreover, small interfering RNA analysis to identify the receptors responsible for mast cell activation by VSV revealed that these receptors reciprocally cooperate to produce anti-viral cytokines and chemokines, inhibiting VSV replication. Our findings suggest that human mast cells produce cytokines and chemokines using several viral recognition receptors, leading to the inhibition of viral replication. These data provide novel information that improves our understanding of the roles of human mast cells in immune responses against viruses.

## Introduction

Mast cells are tissue resident cells derived from hematopoietic precursor cells [1]. Mast cells reside in the tissues closely associated with blood vessels or on the surface of the body, particularly in the skin and mucosal membranes. Thus, in addition to their roles as effector cells in IgE-mediated allergic diseases, mast cells are widely considered to be important innate immune cells [2, 3].

The roles of mast cells in immune surveillance and innate immunity against bacterial pathogens have been well defined [4]. Generally, host innate immune responses against various pathogens are initiated by the recognition of microbial-specific components, such as lipopolysaccharides (LPSs), lipoproteins, flagellins and nucleic acids, by pattern recognition receptors (PRRs). The recognition of microbial components by PRRs results in the coordinated activation of transcription factors, leading to the expression of inflammatory cytokines, chemokines and type I interferons (IFNs). We and others have previously shown that mast cells contribute to innate immune responses against invading bacteria by expressing PRRs, such as TLRs-2, 3, 4, 5, 6, 7 and 9, which respond to specific ligands by inducing the production of cytokines and chemokines or the release of the cell’s granular contents [5–7]. Similarly, innate immune responses against viral infection begin with the recognition of the virus by specific PRRs. Viral nucleic acids are known to be recognized by distinct types of sensors: TLRs, which detect double-stranded (ds) or single-stranded (ss) RNA in the endosome; retinoic acid-inducible gene-I-like receptors (RLRs), which recognize viral RNA in the cytoplasm; and DNA sensors, which detect cytoplasmic viral DNA [8]. The activation of mast cells, including the specific production of anti-viral cytokines such as type I interferon or the release of their granular contents, has been observed upon stimulation with viruses, virus products or poly I:C [9–14]. Additionally, evidence regarding the roles of mast cells in response to viral infections has been accumulating. Orinska et al. [15] reported the functional consequences of mast cell activation in response to viral infection, demonstrating that mast cells stimulated via TLR3 produced chemokines that mediated CD8^+^ T cell recruitment in vivo. Burke et al. [16] demonstrated that poly I:C-exposed or reovirus-infected mast cells recruit NK cells in a CXCL8-dependent manner. Furthermore, the TLR-induced activation of mast cells by LPS or poly I:C enhanced the capacity of mast cells to activate CD8^+^ T cells [17]. More recently, mast cells have been shown to play important roles in host protection against herpes simplex virus 2 (HSV-2) infection [18]. Thus, clarifying the mechanisms of virus recognition that lead to mast cell activation is important for understanding the roles played by mast cells in virus infection because mast cells work not only as innate immune cells for host protection, but also as exaggerators of allergic diseases associated with virus infection.

Although the knowledge regarding how mast cells are activated by viruses or virus products is limited, we previously demonstrated that mouse mast cells express several recognition receptors for viral products and play important roles in early anti-viral cytokine responses via the recognition of viral products by these receptors [19]. Because the differences between human and rodent mast cells reside not only in their phenotypic differences but also in the expression of innate immune receptors such as PRRs and RLRs, in this study, we further analyzed the anti-viral responses of human mast cells using a PCR array system. We found that 2′-5′ oligoadenylate synthetase (OAS), which also functions as a virus recognition receptor by activating the latent form of RNase L, leading to viral RNA degradation, was also responsible for the activation of human mast cells by the virus, collaborating with RLRs and TLR3.

## Materials and methods

### Cells

The Laboratory of Allergic Diseases 2 cell line (LAD2), which was isolated from the bone marrow of a patient with mast cell leukemia, was a gift from Dr. Arnold Kirshenbaum (National Institutes of Health, National Institute of Allergy and Infectious Diseases, Bethesda, MD, USA). The LAD2 cells were cultured at 37 °C in StemPro-34 serum-free medium (Gibco by Life Technologies, Tokyo, Japan) supplemented with StemPro-34 nutrient (Gibco), l-glutamine (200 mM) (Gibco), penicillin (100 U/ml) and streptomycin (100 μg/ml) (Meiji Seika Pharma Co., Ltd, Tokyo, Japan), as well as recombinant human stem cell factor and cytokines (100 ng/ml) (PeproTech, Rocky Hill, NJ, USA).

### Virus infection

Vesicular stomatitis virus (VSV) was kindly provided by Drs. K. Honda and H. Yanai (The University of Tokyo). LAD2 cells (1 × 10^6^ cells/ml) were stimulated in medium without antibiotics with VSV at the indicated multiplicities of infection (MOIs) for 1 to 24 h at 37 °C. To analyze the degranulation of the cells, LAD2 cells were resuspended at 1 × 10^6^ cells/ml in Tyrode’s buffer containing 0.1 % BSA and stimulated with VSV at the indicated MOIs. The release of β-hexosaminidase was measured as previously described [5]. Degranulation was expressed as the percent release of β-hexosaminidase [(OD: stimulated-un-stimulated/OD: total lysate-un-stimulated) × 100 %].

### Quantitative PCR

Total RNA was extracted from the cells using an RNAspin Mini kit (GE Healthcare, Buckinghamshire, UK) and treated with DNase I (GE Healthcare) and an RNeasy Plus Mini kit (Qiagen, Hilden, Germany). Complementary DNA was synthesized using SuperScript™ II reverse transcriptase (Invitrogen, Tokyo, Japan) with random primers. Quantitative PCR was performed using the TaqMan method with an ABI 7500 System (Applied Biosystems, Piscataway, NJ, USA). The mRNA levels were normalized to β-actin gene expression and expressed as the fold-induction relative to uninfected cells.

### PCR array analysis

The Human Antiviral Response RT^2^ Profiler PCR Array (Qiagen) was used to profile the expression of 84 key genes involved in the innate antiviral immune response according to the manufacturer’s instructions.

### Measurement of cytokines and chemokines

LAD2 cells (1 × 10^6^/ml) were stimulated with the indicated concentrations of virus for 24 h. The IFN-α and IFN-β concentrations in the supernatants were determined using ELISA kits according to the manufacturer’s instructions (PBL Assay Science, Piscataway, NJ, USA). The IL-6, CCL5 (RANTES), CXCL10 (IP10), CXCL11 and IL-15 concentrations in the supernatants were also determined using ELISA kits according to the manufacturer’s instructions (R&D Systems, Inc. Minneapolis, MN, USA).

### Western blotting

LAD2 cells were untreated or infected with VSV (MOI = 100 or 300) for 24 h. After stopping the reaction by adding ice-cold PBS, sample buffer containing SDS was directly added to the pellets, followed by brief sonication. Then, the lysates were subjected to SDS-PAGE and transferred onto polyvinylidene difluoride membranes (Merck Millipore, Tokyo, Japan). The membranes were analyzed by immunoblotting with anti-MDA5, anti-RIG-I (both from Cell Signaling Technology, Danvers, MA, USA), anti-TLR3 (Acris Antibodies, San Diego, CA, USA), anti-OAS2 (OriGene Technologies, Inc., Rockville, MD, USA) or anti-β-actin antibody (BioLegend, San Diego, CA, USA), followed by the respective HRP-conjugated secondary anti-immunoglobulin antibodies (GE Healthcare). The membranes were developed using Luminata™ Forte Western HRP substrate (Merck Millipore).

### Small interfering RNA (siRNA) treatment

LAD2 cells were transfected with control or specific siRNA for *rig*-*I*, *mda5*, *tlr3*, *oas1*, *oas2* and *oas3* (Applied Biosystems) using the transfecting system NEON (program 16). The individual gene silencing efficiency was determined by quantitative PCR 24 h after transfection, and then the cells were used for experiments.

### Measurement of virus titer

LAD2 cells were infected with VSV (MOI = 10) 24 h after the corresponding siRNA transfection. The titers of VSV in the supernatant of LAD2 cells 24 h after VSV infection were determined by counting the plaque forming units (PFUs) using Vero cells. Briefly, serially diluted supernatant of the LAD2 cells was incubated with Vero cells for 1 h. After removing the supernatant, the Vero cells were covered with 3% methylcellulose and incubated for another 24 h before counting the formed plaques.

### Statistical analysis

A nonparametric Mann–Whitney *U* test was used. Values of *p* < 0.05 were considered statistically significant.

## Results

### Anti-viral responsive gene analysis of human mast cells upon virus infection using quantitative PCR array

Because we previously demonstrated that murine mast cells can respond to virus infection to produce type I interferon and several chemokines by up-regulating the RLRs, quantitative PCR array analysis of the innate immune response against viruses was performed in LAD2 cells after VSV infection. We found that several anti-viral responsive genes, including cytokines, chemokines and RLRs, were up-regulated 24 h after VSV (MOI = 100) infection (Table 1). Among these genes, we first identified the up-regulation of OAS genes that function as intracellular dsRNA sensors in human mast cells by activating the synthesis of 2′-5′-linked oligoadenylates (2-5As). Then, 2-5As activate the latent forms of RNase L, which leads to viral RNA degradation, thus acting as an anti-viral mechanism.

**Table 1:** Anti-viral responsive gene analysis of human mast cells upon virus infection using quantitative PCR array

Symbol	Fold change
CCL5	2.1425
CXCL10	1,360.225
CXCL11	509.8465
DDX58 (RIG-I)	14.1962
IFIH1 (MDA5)	9.1723
IFNA1	312.5437
IFNB1	623.459
IL15	3.2159
OAS2	52.4045
TLR3	370.1766

LAD2 cells were stimulated with the indicated concentrations of VSV (MOI = 100) for 24 h. The Human Antiviral Response RT^2^ Profiler PCR Array was used to profile the expression of 84 key genes involved in the innate antiviral immune response according to the manufacturer’s instructions

### Viral infection induced anti-viral cytokine and chemokine responses in human mast cells

**Fig. 1:**
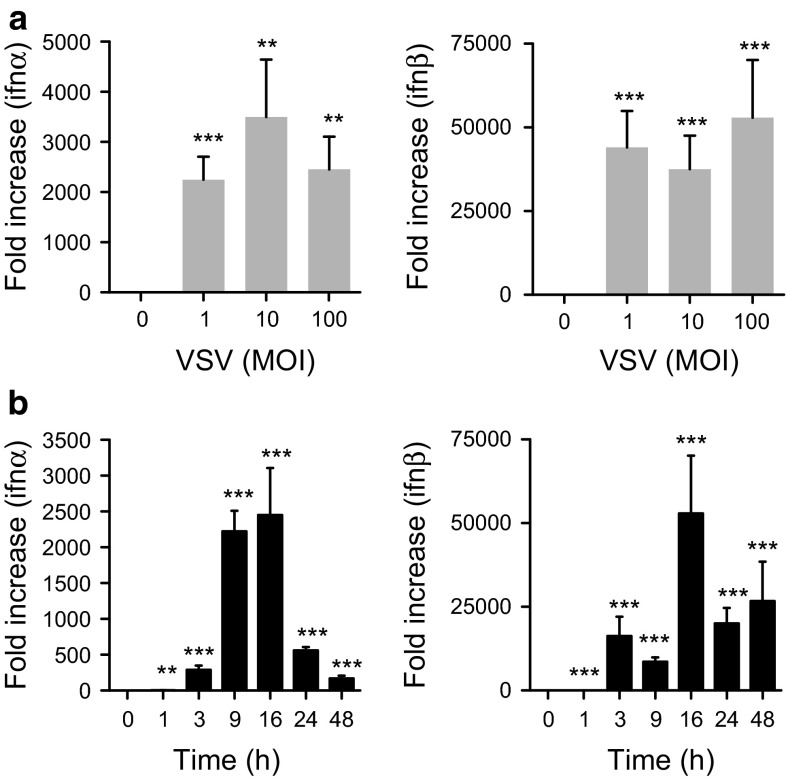
Human mast cell line LAD2 cells are responsive to virus infection by inducing anti-viral type I interferons. LAD2 cells were stimulated with the indicated concentrations of VSV for 24 h (**a**) or with VSV (MOI = 100) for the indicated times (**b**). The levels of mRNA for IFN-α and IFN-β were determined by quantitative PCR. The *columns* present the mean ± SD of 3 separate experiments. *p < 0.05; **p < 0.01; ***p < 0.001 compared with un-stimulated cells

**Fig. 2:**
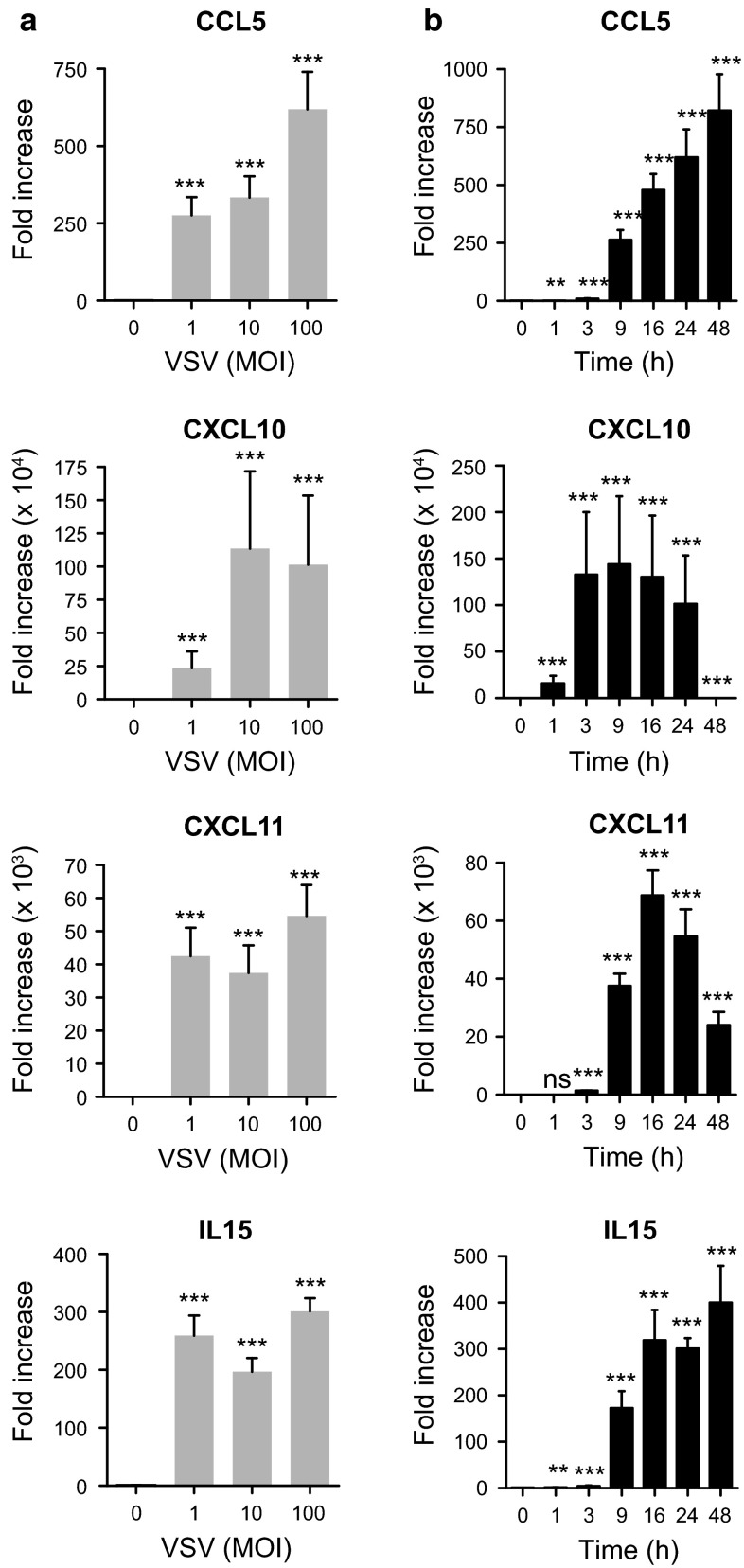
Viral infection directly causes anti-viral chemokine production in a human mast cell line. LAD2 cells were stimulated with various concentrations of VSV for 24 h (**a**) or with VSV (MOI = 100) for the indicated times (**b**). The levels of mRNA for CCL5, CXCL10, CXCL11 and IL-15 were determined by quantitative PCR. The *columns* present the mean ± SD of 3–5 separate experiments. *p < 0.05; **p < 0.01; ***p < 0.001 compared with un-stimulated cells

**Fig. 3:**
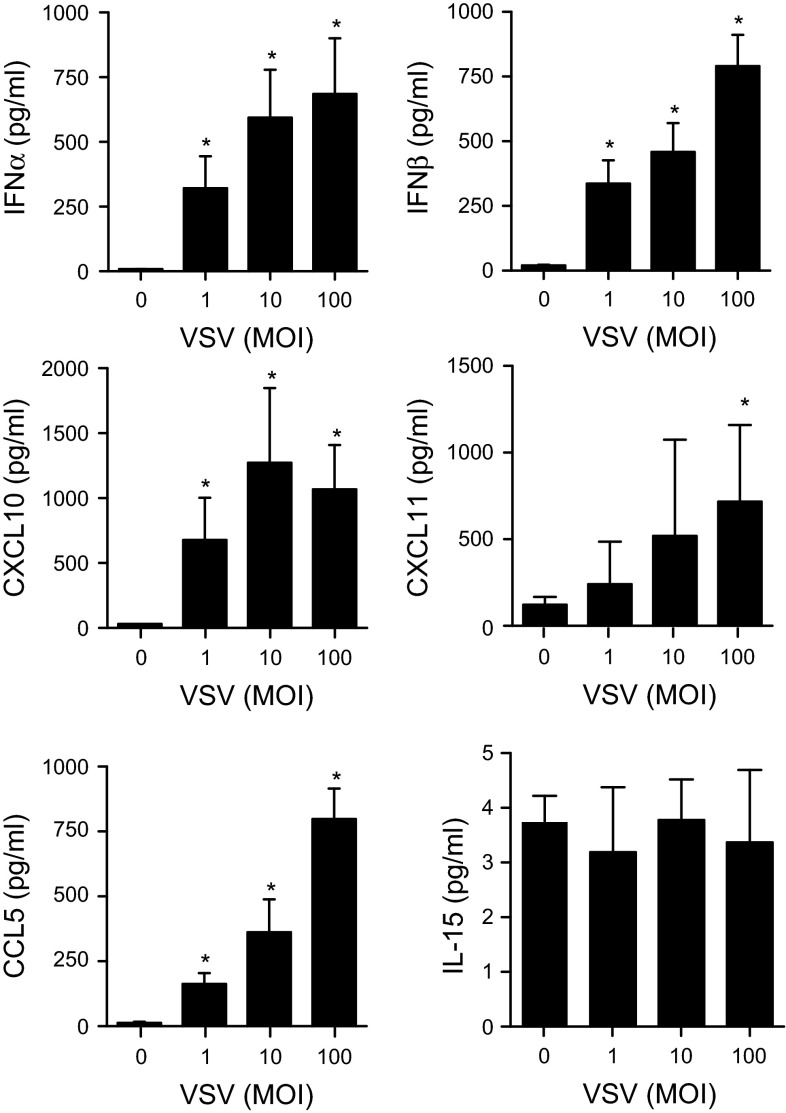
Viral infection causes antiviral cytokine production at the protein level by human mast cell line LAD2 cells. The production of IFN-α, IFN-β, CCL5, CXCL10, CXCL11 and IL-15 upon stimulation with the indicated concentrations of VSV for 24 h was measured by ELISA as described in the “Materials and methods” section. The *columns* present the mean ± SD of 3 separate experiments. *p < 0.05; **p < 0.01; ***p < 0.001 compared with un-stimulated cells

To confirm the PCR array results, first, we cultured LAD2 cells with different doses of VSV for 1–48 h. VSV (MOI = 1–100) infection resulted in both IFN-α and IFN-β induction in mast cells (Fig. 1a), and the peak time of the induction was approximately 16 h after infection (Fig. 1b). We also confirmed anti-viral chemokine expression by LAD2 cells upon viral infection. We observed CCL5 (RANTES), CXCL10 (IP-10), CXCL11, and IL-15 induction in LAD2 cells upon VSV infection (MOI = 1–100) (Fig. 2a). The time course analysis revealed that the peak expression times of CXCL10 and CXCL11 were similar to type I IFNs; in contrast, the induction of CCL5 and IL-15 seemed to be late compared with that of the other chemokines (Fig. 2b). Except for IL-15, the production of these cytokines and chemokines was also detectable at the protein level when measured by ELISA (Fig. 3).

### VSV infection did not induce human mast cell degranulation

**Fig. 4:**
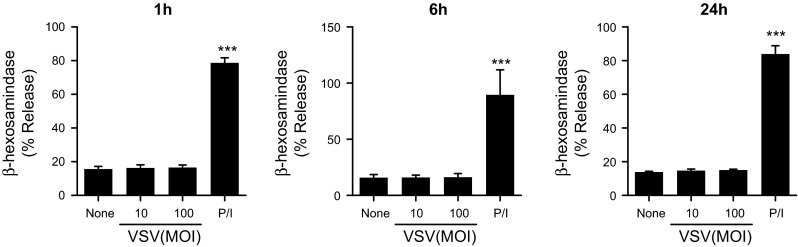
LAD2 cells do not degranulate upon VSV infection. LAD2 cells were stimulated with the indicated concentrations of VSV for 1 to 24 h. β-hexosaminidase release was measured as described in the “Materials and methods” section. PMA/ionomycin was used as a positive control. The *columns* present the mean ± SD of 3 separate experiments. *p < 0.05; **p < 0.01; ***p < 0.001 compared with un-stimulated cells

Because mast cell activation is often associated with degranulation, we examined whether VSV infection leads to mast cell degranulation. We could not detect significant β-hexosaminidase release after 1–24 h of VSV infection (Fig. 4). This result was not consistent with that obtained in murine mast cells, where we observed slight but significant β-hexosaminidase release from murine mast cells at 6 h, but not at 1 h, after VSV infection [19].

### LAD2 cells expressed several RLRs and OAS that were up-regulated by VSV infection

**Fig. 5:**
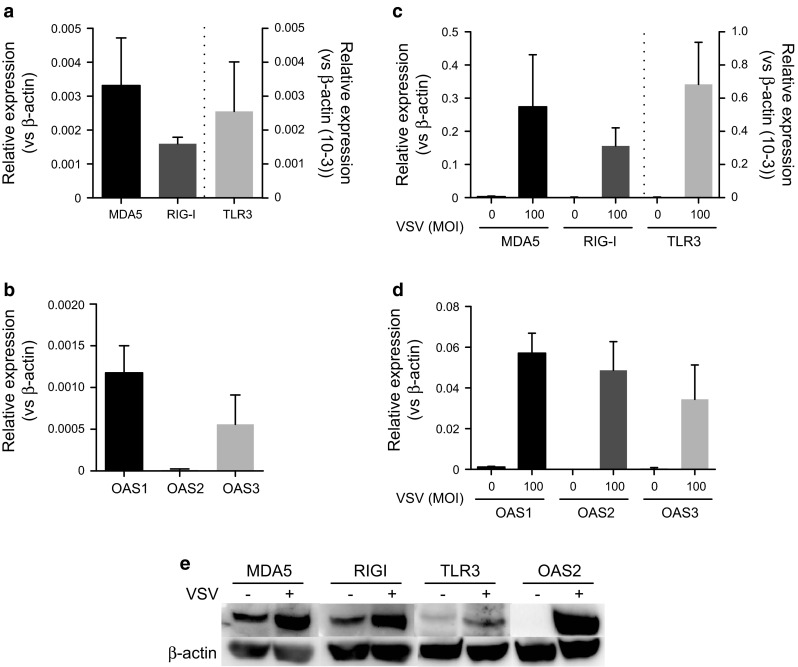
LAD2 cells express several OAS proteins, in addition to RLRs, and their expression levels are up-regulated by viral infection. The mRNA levels of MDA5, RIG-I, and TLR3 in (**a**) un-stimulated LAD2 or (**b**) LAD2 cells stimulated with VSV (MOI = 100) for 24 h were quantified by real-time PCR and expressed as relative expression levels based on the β-actin levels. The mRNA levels of OAS1–3 in un-stimulated LAD2 cells (**c**) or LAD2 cells stimulated with VSV (MOI = 100) for 24 h (**d**) were quantified by real-time PCR and expressed as relative expression levels based on the β-actin levels. (**e**) The protein levels of these receptors were analyzed by Western blotting. Equal protein loading in each *lane* was confirmed by reprobing the same membrane with anti-β-actin. The *columns* present the mean ± SD of 3 experiments. *p < 0.05; **p < 0.01; ***p < 0.001 compared with the un-stimulated cells

Because we found that **s**everal intracellular receptors against dsRNA, including RLRs and OAS, were up-regulated in LAD2 cells upon VSV infection, we further examined whether these receptors play roles in the recognition and activation of human mast cells by VSV. First, we measured the mRNA levels of MDA5, RIG-I, TLR3, OAS1, OAS2, and OAS3 in un-stimulated mast cells by real-time PCR. TLR3 expression was overwhelmingly low compared with that of the other receptors (Fig. 5a). The expression of all of the receptors was markedly increased by VSV infection, with a significant difference (MOI = 100) after 24 h (Fig. 5b). The four OAS genes [OAS1, 2, 3 and OAS-like (OASL)] were identified in humans and have been mapped to chromosome 12. Among these genes, OAS1–3 have considerable homology to each other. The expression of OAS1 and OAS3 was abundant in un-stimulated mast cells (Fig. 5c). Although the expression of OAS2 was low in un-stimulated mast cells compared with OAS1 and 3, its expression level was drastically up-regulated upon VSV infection (Fig. 5d). We also analyzed the expression of MDA5, RIG-I, TLR3 and OAS2 at the protein level by Western blotting. Consistent with the mRNA results, increased expression of MDA5, RIG-I and OAS2 was observed and TLR3 expression was less robust at the protein level upon virus infection (Fig. 5e).

### siRNA-mediated knockdown of RNA sensors partially inhibited the cytokine and chemokine responses of human mast cells to VSV infection and affected their replication

**Fig. 6:**
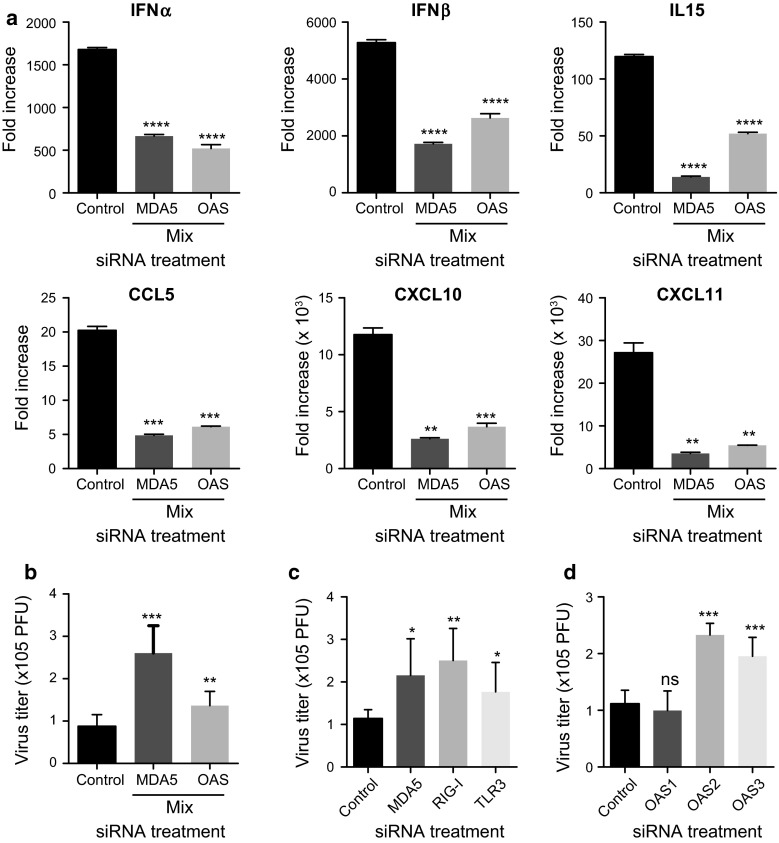
Several virus recognition receptors reciprocally contribute to anti-viral cytokines and chemokines and to VSV replication. Small interfering RNAs were used to knockdown endogenous MDA5, RIG-I and TLR3 or OAS 1-3 as described in the “Materials and methods” section. The cells were stimulated with VSV 24 h after the corresponding siRNA transfection. The levels of IFN-α, IFN-β, CCL5, CXCL10, CXCL11 and IL-15 were determined by real-time PCR 24 h after VSV infection (**a**). Virus replication was examined using Vero cells infected with the supernatants of LAD2 cells that were stimulated with VSV after the corresponding knockdown of receptors as described in the “Materials and methods” section (**b**, **c**, **d**). The *columns* present the mean ± SD of 3 separate experiments. *p < 0.05; **p < 0.01; ***p < 0.001 compared with the control siRNA-treated cells

To investigate whether RNA sensors are responsible for the recognition and activation of human mast cells during viral infection, we used specific siRNAs to knockdown the respective RNA sensors and examined the anti-viral cytokine and chemokine responses. The specific knockdown of each RNA sensor by the corresponding siRNA was confirmed 24 h after siRNA transfection (data not shown). The cells were stimulated by VSV infection, and their cytokine and chemokine expression was measured by real-time PCR. Although we observed significant but minimal inhibition of cytokine-chemokine induction by the knockdown of individual receptors, the simultaneous knockdown of MDA5, RIG-I and TLR3, along with the knockdown of OAS1–3, significantly inhibited the cytokine and chemokine responses to VSV infection (Fig. 6a). Additionally, we examined the titer of VSV by counting plaque forming units (PFUs) using Vero cells infected with the supernatant of VSV-infected LAD2 cells. Consistent with the results of the cytokine and chemokine analysis, the knockdown of MDA5, RIG-I and TLR3 and OAS1–3 significantly increased VSV replication, suggesting that mast cells exert direct anti-viral responses via the recognition of viral products using these receptors (Fig. 6b). Further investigation suggested that all of these receptors, except OAS1, were responsible for the anti-viral responses of human mast cells infected with VSV (Fig. 6c).

## Discussion

We previously demonstrated that murine bone marrow-derived mast cells expressed several viral recognition receptors and responded to viruses via these receptors by producing anti-viral cytokines and chemokines [19]. Mast cells are tissue resident cells derived from hematopoietic stem cells in the bone marrow, which enter the peripheral blood as immature cells and differentiate into mature mast cells under the influence of the surrounding microenvironment [1]. Mast cells display various phenotypes; in particular, these cells differentially express protease in their granules according to the host source and to the associated tissue or organ. Mast cells also possess a complement of PRRs that differentially respond against pathogen-associated molecular patterns (PAMPs) [20–22]. Several reports have defined the differential usage of PRRs between human and murine mast cells for the recognition of PAMPs. Thus, in this study, we aimed to identify the virus recognition receptors and their roles in human mast cell activation during virus infection.

We performed PCR array analysis of human mast cells upon VSV infection to identify the genes that are regulated during this infection. As expected, we observed the up-regulation of several genes encoding anti-viral cytokine, chemokines and receptors involved in the recognition of viruses (Table 1). The low expression levels of TLR3 compared with other RLRs were similar in murine mast cells; however, unlike in murine mast cells [19, 23], the lack of TLR3 in human mast cells slightly but significantly reduced the cytokine and chemokine responses and affected VSV replication (Fig. 6), suggesting that TLR3 is also responsible for the recognition of viral products during VSV infection in humans. Although TLR3 has repeatedly been assumed to play an important role in the activation of human and rodent mast cells by poly I:C and several viruses [9, 24], this phenomenon varied among the populations of examined mast cells [25]. Because TLR3-mediated mast cell activation is required for the recruitment of CD8^+^ T cells to the site of viral infection in some situations [15, 26], TLR3-mediated systems provide different contributions depending on the type of virus and on the route of infection. Although we could detect increased TLR3 expression in VSV-stimulated human mast cells, no significant differences in the expression of other TLRs were observed (data not shown).

RLRs are broadly expressed in most tissues, and their expression is typically maintained at low levels in resting cells but greatly increased after IFN exposure and viral infection [27, 28]. We observed a drastic increase in the RNA sensors RIG-I and MDA5 in VSV-stimulated human mast cells. A similar up-regulation of the mRNA and protein levels of RLRs, including multiple interferon-stimulated genes, was also observed in human mast cells in response to antibody-enhanced Dengue virus or Sendai virus infection [25, 29]. VSV, which is a member of the Rhabdovirus family, contains a single-stranded negative-sense RNA genome. The mechanism by which the innate immune system detects VSV has been thoroughly investigated [30–34]; RIG-I mediates a specific response to VSV, as well as to many other negative-strand viruses, such as influenza and Sendai virus, and some positive-strand RNA viruses, such as Japanese encephalitis virus [35]. However, the previous results of our group or others using mouse mast cells have suggested that the responses against VSV were not solely mediated by RIG-I [19, 36, 37]. Our present results suggest that human mast cells responded to VSV via MDA5 and TLR3, in addition to RIG-I (Fig. 6a). Thus, the dependency on different RLRs to recognize different viruses appears to be variable among cells, and the various receptors may provide different contributions depending on the route of infection.

The present study demonstrated for the first time that human mast cells express and up-regulate OAS upon VSV infection (Table 1; Fig. 5). OAS proteins are a family of anti-viral restriction factors that target a wide range of RNA and DNA viruses [38]. These proteins function as intracellular dsRNA sensors that undergo a conformational change upon binding to dsRNA and that are activated to synthesize 2’-5’-linked oligoadenylates (2-5As). Then, 2-5As activate the latent form of RNase L, which leads to viral RNA degradation [39]. In recent years, the contributions of RNase L and OAS to innate immunity have become increasingly apparent [40]. Because the small RNA cleavage products produced by RNase L during viral infections can reportedly signal to the RIG-I receptor [41], our finding that the anti-viral effect mediated by OAS-RNase L was either dependent on or independent of the RIG-receptor system in our studies is notable. siRNA knockdown experiments indicated that OAS knockdown significantly reduced cytokine and chemokine production and virus replication; however, the effect was less complete than the knockdown effects of cells treated with siRNAs for RLRs and TLR3, suggesting that the RIG-I receptor could recognize also the degraded RNA independent of the OAS system (Fig. 6).

The identified OAS genes in humans have been named OAS1, OAS2, OAS3 and OASL (OAS-like) [42]. OAS1–3 have considerable homology to each other, with OAS1, OAS2 and OAS3 encoding one, two and three “OAS” domains, respectively [40]. However, the roles of OAS1–3 in individual viral infections have not been fully investigated [42]. In the PCR array, although we primarily focused on OAS2, we also investigated the expression of OAS 1 and 3. Because the relative expression of OAS2 in un-stimulated human mast cells was low compared with that of OAS1 and OAS3, the drastic increase in OAS2 expression upon VSV infection was obvious. The knockdown experiments for each OAS revealed that OAS2 and 3, but not OAS1, were responsible for the anti-viral effects of human mast cells.

Most pathogens appear to induce lipid mediator and cytokine secretion but not degranulation [7]. We found that VSV infection did not induce β-hexosaminidase release in human mast cells, which is also consistent with previous reports [13, 43] but differs from the results obtained with mouse mast cells [19], in which we observed the degranulation of mouse mast cells after 6 h, but not after 1 h, of VSV infection in the absence of MDA5, RIG-I, PKR and TLR3 [19]. Further studies are required to determine the mechanisms of virus recognition that lead to mast cell degranulation, as well as the differences between human and mouse mast cells.

The induction of type 1 IFNs, including IFN-α and IFN-β, by viruses and other pathogens is of crucial importance for innate immunity [44]. In addition, we observed the up-regulation of several chemokines in human mast cells upon VSV infection (Table 1, Figs. 2, 3). CXCL10, which is a chemokine associated with type 1 T cell responses, is highly expressed in virus-infected cells and is involved in regulating the migration of effector T cells at the sites of inflammation through binding to the CXCR3 receptor [45]. Although the actual role of CXCL11 in viral infections remains unclear, the peak level of CXCL11 mRNA coincides with the peak of viremia and the CXCL11 protein was reported to inhibit viral growth [46]. CXCL11 also directly inhibits respiratory adenovirus serotypes Ad3 and Ad5 [47]. CCL5 is a chemokine and an important inflammatory mediator; CCL5 regulates diseases due to viral infection by signaling via CCR5 on CD8(+) T cells and modulates anti-viral activities [48]. Although we could not detect protein production, a significant increase in IL-15 mRNA was observed upon VSV infection. IL-15 is a well-known cytokine crucial for the development and function of NK cells, which are described as guardians for the detection and clearance of virus-infected cells [49].

Thus, the present findings strengthen the role of mast cells as key responders of the immune response during the early stages of viral infection via their ability to directly recognize and quickly respond to a virus by the rapid production of anti-viral cytokines and chemokines using RLRs and OAS-RNase L, in addition to TLR3. Notably, both allergens and microbial antigens can trigger mast cell activation, and allergic or autoimmune diseases, where mast cells play important roles in their pathogenesis, are often exacerbated by viruses such as rhinovirus [50, 51]. Thus, this knowledge could potentially be exploited therapeutically to modulate host antiviral immunity by targeting these pathways in mast cells.
